# Identification of Antimicrobial Peptides from *Nibribacter radioresistens*, a UV and Gamma Radiation Tolerant Bacterium

**DOI:** 10.3390/genes16030353

**Published:** 2025-03-19

**Authors:** Sam Woong Kim, Woo Young Bang

**Affiliations:** 1 Agri-Food Bio Convergence Institute, Gyeongsang National University, Jinju 52725, Republic of Korea; 2 Biodiversity Research and Cooperation Division, National Institute of Biological Resources (NIBR), Environmental Research Complex, Incheon 22689, Republic of Korea

**Keywords:** AMPs, antibacterial, cell-free supernatant, genome, transcriptome, peptide

## Abstract

Background: *Nibribacter radioresistens*, a UV and gamma radiation-tolerant bacterium, was reported to have superior antibacterial activities against a variety of pathogenic bacteria through the production of antimicrobial peptides (AMPs), but nothing is known about its AMPs. Methods/Results: In this study, our genomic and transcriptomic data showed that the *N. radioresistens* genome contains 11 *AMP* gene candidates, designated as NB_AMP1 to NB_AMP11, which are expressed differently in logarithmic growth and stationary phase. Moreover, the cell-free supernatant of all *Escherichia coli* DH5α strains containing cloned *AMPs* except for NB_AMP5 and NB_AMP7 exhibited antibacterial activities against both Gram-negative and Gram-positive bacteria such as *E. coli* and *Staphylococcus aureus*. Synthetic AMPs supported the antibacterial activities of cloned *AMPs*, and, in particular, the synthetic NB_AMP2 showed superior antibacterial activities against both *E. coli* and *S. aureus*. Conclusions: Altogether, these results suggest that the *AMP* candidates from *N. radioresistens* may function as antimicrobial peptides, effectively causing cellular lysis through pore formation in the bacterial membrane.

## 1. Introduction

Antimicrobial peptides (AMPs) are receiving great attention as next-generation antimicrobial agents due to their unique properties and effects against a variety of pathogens [1,2,3,4]. For that reason, first, AMPs can target a variety of microorganisms, including bacteria, fungi, viruses, and even cancer cells [5]. These extensive spectral activities are versatile and effective against a variety of infections. Second, AMPs have several mechanisms of action, including destroying microbial membranes, interfering with cell wall synthesis, and inhibiting protein synthesis [1,2,3,4]. Especially, the AMPs that are resistant to proteases can be valuable alternatives in the fight against pathogenic microorganisms that are resistant to existing antibiotics [6]. Lastly, AMPs can improve the body’s natural defense against infection by regulating the immune response [7]. The dual action of these direct antimicrobial activities and immune system support is an important advantage. These properties highlight the potential of AMPs as powerful tools to address the growing challenges of combating infectious diseases and antibiotic resistance.

AMPs with excellent antibacterial activity can be found in various microorganisms, based on the fact that various microorganisms utilize AMPs as a means of self-defense [6]. For example, Microcin J25 is found in *E. coli*, which has antibacterial activity against certain types of bacteria [6]. Nisin is found in *Lactococcus lactis*, a lactic acid bacterium, and is particularly effective against Gram-positive bacteria [6]. Colicins are found in *E. coli*, which, upon secretion from the host, kill non-host *E. coli* strains. Enterocins are found in *Enterococcus* species and are effective against a variety of Gram-positive bacteria [6].

*Nibribacter*, a genus belonging to the family *Hymenobacteraceae* within the phylum *Bacteroidota*, comprises Gram-negative, rod-shaped, and aerobic bacteria, including *N. flagellatus*, *N. koreensis*, *N. ruber*, and *N. radioresistens* [8]. In the previous study, we identified that the cell-free supernatant of the *N. radioresistens*, a red pigmented and UV and gamma radiation tolerant bacterium isolated from seawater and closely related to *N. flagellatus*, exhibited antibacterial activity against various pathogens, and, in particular, experiments using proteinase K and EDTA implied strongly that peptide substances like AMPs may play an important role in the antibacterial activity of the cell-free supernatant [8]. Therefore, in this study, we tried to find AMP candidates through genomic and transcriptomic analyses of *N. radioresistens* and identified several AMPs with excellent antibacterial activity. These results suggest that the AMPs, discovered from the *N. radioresistens*, may be used as antibacterial substances that can replace existing antibiotics.

## 2. Materials and Methods

### 2.1. Medium and Bacterial Strains Employed for This Study

The media used in this study were R2A, TSB, and LB (Sigma-Aldrich, St. Louis, MO, USA). If required, the solid medium was made by supplementation with 1.5% agar. An *N. radioresistens* strain with the culture number, NIBRBAC000499663, was obtained from the National Institute of Biological Resources (Incheon, Republic of Korea) and was cultured for 24 to 48 h at 25 °C in R2A medium. *E. coli* ATCC 10536 and *S. aureus* ATCC 6538 were purchased from KCTC (Korean Collection for Type of Cultures, Daejeon, Republic of Korea) and were incubated at 37 °C.

### 2.2. N. radioresistens Genome Sequencing

Genomes were isolated from *N. radioresistens* using a genome preparation kit (Wizard Genomic DNA purification kit, Promega, Madison, WI, USA), and 5 μg of purified genome was used for library preparation. A SMRTbell library was constructed according to the manufacturer’s protocol for the SMRTbell™ Template Prep Kit 1.0 (PN 100-259-100, Pacific Biosciences, Menlo Park, CA, USA). Small DNA fragments of less than 20 kb of SMRTbell template were removed using a Blue Pipin Size Selection system (Sage Science, Beverly, MA, USA) for the large-insert library. The constructed library was subjected to quality control using the Agilent 2100 Bioanalyzer (Agilent Technologies, Santa Clara, CA, USA). Sequence analysis oligomers were annealed to the SMRTbell template, and then DNA polymerization was carried out using the DNA/Polymerase Binding kit P6. This polymerase-SMRTbell-adaptor complex was spotted into a SMRT cell. The SMRTell library was sequenced with 1 SMRT cell using C4 chemistry (DNA sequencing Reagent 4.0) with the MagBead OneCellPerWell v1 Protocol (Insert Sizes 20 kb, movie time 1 × 240 min). Images were recorded in the SMRT cell for 240 min using the Pacific Biosciences (PacBio RS II) sequencing platform. This resulted in 116,823 and 1,148,362,395 base pairs of long reads following subread filtering.

De novo assembly was performed using a hierarchical genome assembly process (HGAP, Version 2.3) workflow containing consensus policing with Quiver (https://github.com/lukeping/GenomicConsensus) (accessed on 12 March 2024). As the estimated genome size was 4,159,177 bp and the average coverage was 86×, we performed error correction based on the longest of about 30× seed bases (150,005,170 bp), with the rest being shorter reads, and then assembled this with error-corrected reads. As a result of the HGAP process, 4,143,062 bp N50 contig and 4,143,062 bp total contig lengths were obtained by the polishing process. Finally, since bacterial genomes and plasmids are typically circular forms, each contig was examined using MUMmer 3.5 (https://mummer4.github.io/) (accessed on 29 March 2024), and one of the self-similar ends was trimmed for manual genome closure.

Putative gene coding sequences (CDSs) from assembly contigs were identified using Glimmer v3.02 (https://ccb.jhu.edu/software/glimmer/index.shtml) (accessed on 1 April 2024), and open reading frames (ORFs) were obtained. These ORFs were explored using Blastall alignment (http://www.ncbi.nlm.nih.gov/books/NBK1762) (accessed on 3 April 2024) in a non-redundant protein database (nr). GO annotation was assigned to each ORF using Blast2GO software (Version 5.2) through maximum hit analysis of BLAST results. Additionally, ribosomal RNAs and transfer RNAs were predicted using RNAmmer 1.2 (https://services.healthtech.dtu.dk/services/RNAmmer-1.2/)(accessed on 9 April 2024) and tRNAScan-SE 1.4 (https://lowelab.ucsc.edu/tRNAscan-SE/) (accessed on 11 April 2024).

### 2.3. Bioinformatic Analysis for Identification of AMPs from the N. radioresistens Genome

Peptides with a similarity (identities/positives) of more than 50% to existing AMPs or possessing a signal sequence cleaved by an endopeptidase were selected as AMP candidates using CAMP (http://www.camp.bicnirrh.res.in/campHelp.php) (accessed on 15 April 2024) or SignalP 5.0 (https://services.healthtech.dtu.dk/services/SignalP-5.0/) (accessed on 17 April 2024), respectively. Their amino acid and nucleotide sequences were subsequently used for peptide synthesis and gene cloning, respectively.

### 2.4. Exploration of AMPs Gene Expression Through Transcriptomic Analysis

Total RNA extraction was performed using the AccuPrep^®^ Bacterial RNA Extraction Kit (Bioneer, Daejeon, Republic of Korea). RNA purity was measured using 1 μL of total RNA in a NanoDrop1000 spectrometer (Thermo Fisher Scientific, Waltham, MA, USA). The measured RNA Integrity Number (RIN) value was checked using the Agilent 2100 Bioanalyzer (Agilent Technologies) to confirm the quality of the total RNA. A Nugen Universal Prokaryotic RNA-seq kit (Part Number 0363-32, NuGEN Technologies, San Carlos, CA, USA) was used for the fabrication of total RNA sequencing libraries, which were carried out according to the manufacturer’s protocol. Total RNA (300 ng) was used to synthesize first and second strand cDNA with selective primers. Fragmentation of 200 bp size was carried out by sonication using a Covaris S220 (Covaris, Woburn, MA, USA) in a microtube. In Strand Selection II, the rRNA was removed according to the bacterial specifications using the AnyDeplete technique with the AnyDeplete probe (Tecan, Mannedorf, Switzerland). The libraries were amplified using PCR, and the amplified amount was confirmed using capacitive electrophoresis (Bioanalyzer, Agilent Technologies). qPCR was performed with SYBR Green PCR Master Mix (Applied Biosystems, Waltham, MA, USA), and RNA sequencing was performed using an Illumina Novaseq 6000 system (Illumina, San Diego, CA, USA).

### 2.5. N. radioresistens AMPs Gene Cloning and Peptide Synthesis

General gene cloning was performed using the method of Sambrook et al. (1989) [9]. Candidate *AMPs* obtained through the analysis of the genome and transcriptome were designed and synthesized with primers for PCR amplification (Table 1; Genotech, Daejon, Republic of Korea). After amplifying each *AMP* gene using this primer set and PCR PreMix (AcuPower PCRMix, Bioneer), gel extraction was performed. Eluted DNA fragments were ligated to the T-easy vector (Promega) and transformed into *E. coli* DH5α (Invitrogen, Carlsbad, CA, USA) using the heat-shock method using calcium chloride. Cloned *AMPs* were identified using restriction enzyme cleavage and nucleotide sequencing (Applied Biosystems).

Amino acid sequences in regions with homology to predicted AMPs were obtained, and these sequences were synthesized as artificial peptides (Table 2; Cosmogenetech, Seoul, Republic of Korea).

### 2.6. Evaluation of Antibacterial Activity of Cell-Free Supernatants and Synthetic Peptides

The cell-free supernatant of all *E. coli* DH5α strains containing cloned *AMPs* was collected after 24 h of culture and filtered through a 0.2 μm syringe (mixed cellulose esters (MCE), Merck, Darmstadt, Germany), and finally the cell-free supernatant was used for the evaluation of antibacterial activity. The cell-free supernatant from the *E. coli* DH5α, harboring only the plasmid vector without the *NB_AMP* genes, was confirmed as a negative control to have very little effect on antibacterial activities. The synthesized AMPs were suspended at a concentration of 10 mg/mL in distilled water and then used for antibacterial activity. The antibacterial activity against the Gram-negative bacterium, *E. coli* (EC; ATCC 10536), and the Gram-positive bacterium, *Staphylococcus aureus* (SA, ATCC 6538), was analyzed using the microtiter plate method and expressed as minimal inhibition concentration (MIC); the bacteria pre-cultured overnight (O/N) were cultured at 10^6^ CFU/mL with the cell-free supernatant or the synthetic peptides, the reaction mixtures were added to the microtiter plate, and finally the reactivity was observed by O/N culture at 37 °C.

### 2.7. Statistical Analysis

The collected data were analyzed using the PROC ANOVA procedure of the SAS program (ver. 9.2; SAS Institute Inc., Cary, NC, USA). Mean values that differed at the level of 5% significance were verified using Duncan’s multiple range test (DMRT).

## 3. Results and Discussion

### 3.1. The Nibribacter radioresistens Genome Includes Various AMP Genes

Our previous study showed that the cell-free supernatant of *N. radioresistens* has high antimicrobial activity against various pathogenic microorganisms, and it was suggested that peptide-based antimicrobial substances, that is, AMPs, may importantly mediate the antimicrobial activity of the culture supernatant [8]. Therefore, in this study, genome analysis was performed to identify the antimicrobial peptide (*AMP*) genes of *N. radioresistens*.

**Figure 1:**
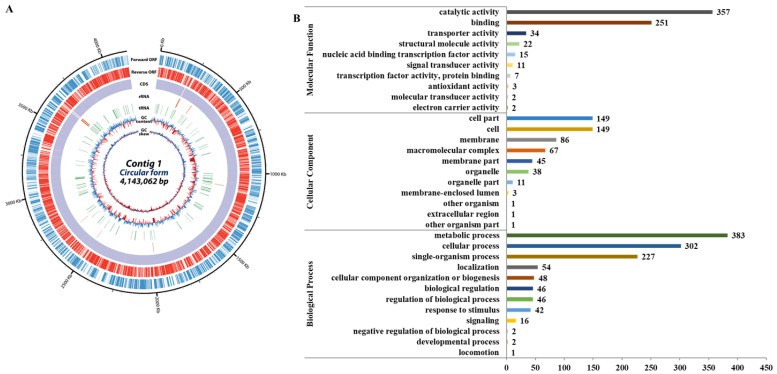
Results of *N. radioresistens* genomic analysis. (**A**) Overall features of the genome. The outer scale indicates the coordinates in base pairs. The open reading frames (ORFs) are shown on the first two rings; the first ring (blue) is the forward ORF, and the second ring (red) is the reverse ORF. The third and fourth circle shows the ORFs which are colored by gene annotation; the third ring is the forward ORF, and the fourth ring is the reverse ORF. The fifth and sixth circles show rRNA (green) and tRNA genes (orange). In the next circle, the GC content shows whether the GC ratio of the DNA sequence is higher (purple) or lower (deep yellow) than the average. The innermost circle shows GC skew, with light green indicating negative values and deep orange for positive values. (**B**) Gene ontology classification of transcriptome. The total genes with BLAST matched against the non-redundant protein database were classified into three main GO categories (biological process, cellular component, molecular function) and 33 subcategories. The *y*-axis shows the detailed names of the subcategories and the *x*-axis indicates the number of genes in the same category.

The *N. radioresistens* genome consists of a single chromosome with a total of 4,143,062 bps, and a total of 3848 genes were predicted, of which 2154 were hypothetical proteins (Figure 1A). Gene ontology analysis revealed that genes associated with biological processes, cellular components, and molecular functions totaled 1169, 551, and 704, respectively. The highest one was the metabolic process (383, 32.8%) of the biological process, followed by the catalytic activity (357, 50.7%) of the molecular function (Figure 1B).

**Figure 2:**
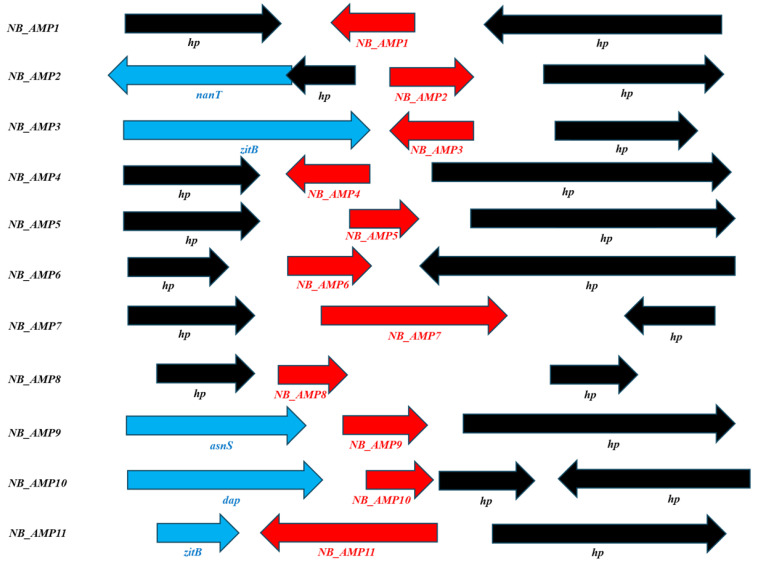
Analyses of predicted AMP neighbor genes. *asnS*; Asparagine-tRNA ligase, *dap*; D-aminopeptidase, *hp*; hypothetical protein, *nanT*; Sialic acid transporter NanT, *zitB*; Zinc transporter ZitB.

Moreover, we selected 321 peptides showing a positivity of 50% or more with existing AMPs through NCBI (https://www.ncbi.nlm.nih.gov/, accessed on 15 April 2024) homology analysis on 2154 genes corresponding to hypothetical or uncharacterized proteins. Among these 321, those having relatively small size or homology with existing AMPs in various regions were primarily selected as a total of 54 hypothetical proteins. Among them, 11 peptides were finally selected based on homology showing more than 50% identities/positives with existing AMPs or on the possession of the signal sequence, cleaved by an endopeptidase, and were designated as NB_AMP1 to NB_AMP11 (Figure 2 and Table 2).

### 3.2. Transcriptomic Results of AMP Candidates from the N. radioresistens Genome

In order to analyze the expression of the *AMP* candidates selected from the *N. radioresistens* genome, transcriptome analysis was performed in the logarithmic growth phase and stationary phase. As shown in Table 3, 3797 genes exhibited the differential expression between the logarithmic growth and stationary phases. Meantime, the 11 *AMP* candidates were identified to be expressed in *N. radioresistens*, and all other genes except for *NB_AMP2* and *NB_AMP11* were found to be highly expressed in the logarithmic growth phase compared with the stationary one (Table 4). In particular, *NB_AMP1*, *NB_AMP3*, *NB_AMP5*, and *NB_AMP8* were observed to be expressed three times higher than in the stationary phase. However, *NB_AMP2* and *NB_AMP11* showed slightly higher values in the stationary phase than in the logarithmic growth phase. In general, it is suggested that genes expressed differently in the logarithmic growth and stationary phases are regulated according to the metabolism and stress response for the adaptation of cells to an environment [10]. In our previous study, the cell-free supernatant of *N. radioresistens* showed the antibacterial activities from the late logarithmic growth phase to the stationary phase [8]. Therefore, these support that the *AMP* candidates with differential expression between logarithmic growth and stationary phases will contribute significantly to the antibacterial activity of the cell-free supernatant of *N. radioresistens*.

### 3.3. Antibacterial Activity of Cloned AMPs from the N. radioresistens Genome

**Figure 3:**
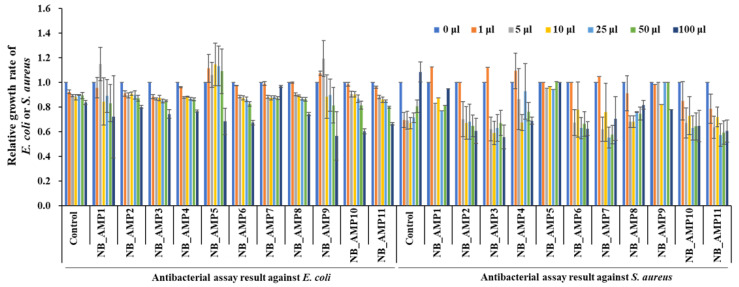
Antibacterial assays of cloned *NB_AMPs*. The cloned *NB_AMPs* were expressed in *E. coli* DH5α, the cell-free supernatants were separated from the *E. coli* DH5α, and then 1 to 100 μL of cell-free supernatants, as indicated at the top of the graph, were applied for antibacterial assays against *E. coli* and *S. aureus*. The *E. coli* and *S. aureus* were adjusted to 10^6^ CFU/mL for the microtiter plate assay.

After the *AMP* candidates are expressed, their signal sequences are expected to be cleaved by endopeptidases to become mature AMPs for their function. Therefore, the PCR-amplified *AMP* candidates were designed to include the entire genes, including *AMP* candidates with their up- and down-stream sequences. DNA fragments containing the PCR-amplified *AMP* genes of *N. radioresistens* were cloned into the T-easy vector, and the antibacterial activity of the cell-free supernatant of the *E. coli* DH5α strains containing cloned *AMPs* was measured against *E. coli* ATCC 10536, a Gram-negative bacterium, and *S. aureus* ATCC 6538, a Gram-positive bacterium. As shown in Figure 3, all except for NB_AMP7 had antibacterial activity against the *E. coli*. In addition, all except for NB_AMP7 and NB_AMP5 exhibited antibacterial activity versus *S. aureus*. Among them, NB_AMP10 and NB_AMP3 showed the highest activity against the *E. coli* and *S. aureus*, respectively.

In order for cloned *AMPs* to exhibit the antibacterial function, they may exhibit efficacy by being delivered to the outside of the cell through a signal sequence or by being released after lysing the cell from the inside. The E protein of bacteriophage phiX174 is a representative antibacterial substance that induces lysis from the bacteria inside [11]. The EcDBS1R6 is known to be a representative AMP that shows its antimicrobial function after its signal peptide is cleaved by an endopeptidase [12]. Noticeably, it is suggested that the NB_AMP3, 6, and 9, predicted to have a signal sequence (Table 2), may be delivered outside the cell via their signal sequence, while the NB_AMP10, predicted to have no signal sequence (Table 2), may be released after lysing the cell from the inside.

### 3.4. Antibacterial Activity of Synthetic AMPs from the N. radioresistens Genome

**Figure 4:**
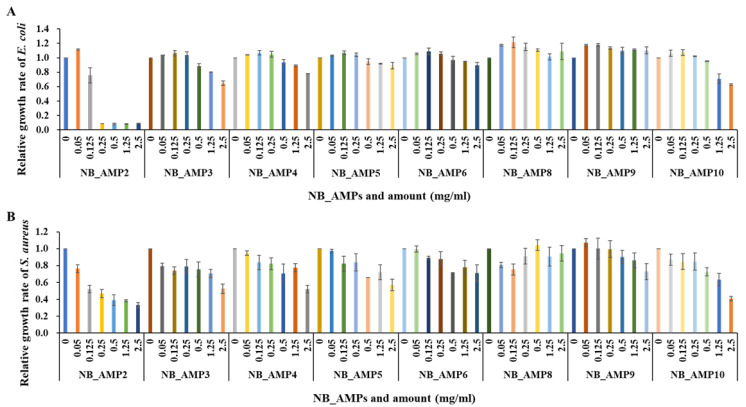
Antibacterial assays of synthetic NB_AMPs. The synthesized NB_AMPs were applied for antibacterial assays against *E. coli* (**A**) and *S. aureus* (**B**). The *x*- and *y*-axes indicate NB_AMP types and concentration and relative growth rate of *E. coli* and *S. aureus*, respectively.

In order to verify the antimicrobial activity of the cell-free supernatant of the strains containing the previously cloned AMPs, only partial regions of the predicted ORFs, corresponding to the homolog of existing AMPs, were chemically synthesized (Table 2) and subjected to antimicrobial analyses. Out of the 11 AMP candidates from the *N. radioresistens* genome, a total of 8 were synthesized successfully, excluding NB_AMP1, NB_AMP7, and NB_AMP11. As a result of evaluating their antibacterial activity, all synthetic AMPs showed antibacterial activity against the *E. coli* (Figure 4A), and, in particular, NB_AMP2, consisting of 14 amino acids in the predicted ORF (Table 2), showed superior antibacterial activity with an MIC50 of 0.17 mg/mL (Table 5). The synthetic AMPs also showed antibacterial activity against the *S. aureus* (Figure 4B), and similarly, the NB_AMP2 showed the best antibacterial activity with an MIC50 of 0.22 mg/mL (Table 5). Compared to existing AMPs, NB_AMP2 exhibited antibacterial activities similar to those of the AMPs. For example, the MIC50 of MGN-1 to MGN-6 against *E. coli* ranges from 2.2 to >128 µg/mL [13], and the MIC50 of P1 to P3 against *E. coli* and *S. aureus* is reported as 256 to >256 and 32 to >256 µg/mL, respectively [14]. These findings underline that the antibacterial activities of AMPs depend on their peptide sequences and structures. Therefore, the antibacterial activity of NB_AMP2 in this study can be further improved through the engineering of its peptide sequences and structures.

## 4. Conclusions

Conventional antibiotics often target specific bacterial components, like cell wall synthesis or protein synthesis, and this allows bacteria to develop resistance by mutating those specific targets [15,16]. However, AMPs offer several potential advantages over the conventional antibiotics in the fight against antibiotic resistance for the following reason: the AMPs frequently disrupt bacterial cell membranes, a broader target that is more difficult for bacteria to alter [17], and this membrane disruption can occur through various modes, making it harder for bacteria to develop resistance to all of them [18]. Particularly, typical AMPs, forming helix structures, effectively cause cellular lysis through pore formation in the bacterial membrane [19].

**Figure 5:**
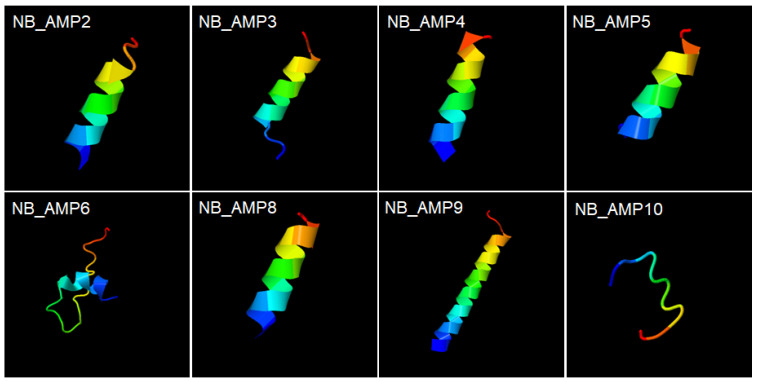
Structural models of synthetic NB_AMPs. The synthesized peptides in Table 2, NB_AMP2, 3, 4, 5, 6, 8, 9, and 10, form helix structures. Colors were used to indicate the direction from the N-terminus to the C-terminus, and the blue and red represent the N-terminus and the C-terminus, respectively. Their structures were predicted by the automated I-TASSER server (https://zhanglab.ccmb.med.umich.edu/I-TASSER/, accessed on 22 February 2025).

In the previous study, the cell-free supernatant of *N. radioresistens* was observed by scanning electron microscopy to cause cellular lysis through pore formation in bacterial membranes, and low molecular weight (<3 kDa) molecules were proved to mediate mainly the antibacterial activity of the cell-free supernatant, implying strongly that the low molecular weight molecules may be peptide substances like AMPs [8]. Interestingly, the structural models for the synthetic NB_AMPs from *N. radioresistens* revealed that except for NB_AMP6 and 10, they form helix structures (Figure 5), which supports that the NB_AMPs with helix structures may effectively cause cellular lysis by damaging the bacterial membrane via pore formation, as did the typical AMPs [19]. Altogether, the results revealed that the AMPs from the *N. radioresistens* genome showed antibacterial activity and supported the reason why the cell-free supernatant of *N. radioresistens* showed antibacterial activity [8].

Even though AMPs are known to have a unique mechanism different from many conventional antibiotics and thus have a low chance of developing resistance, it is known that when bacteria are exposed to AMPs for a long period of time, some bacterial populations acquire resistance to the AMPs through various strategies. For example, bacteria can interfere with the binding of AMPs by reducing the negative charge on their membrane or increasing the density of the cell wall [20]. The resistance can also be acquired by activating a pump that transports AMPs to the outside of the bacteria and by degrading AMPs through specific enzymes [21]. In addition, the AMPs have potential limitations in their clinical application, such as non-specific antibacterial action against beneficial bacteria as well as pathogens, potential toxicity to host cells, and difficulties in large-scale production [22]. To solve these limitations, it is possible to adjust the positive charge of peptides to reduce the non-specific action of AMPs, reduce the resistance by using AMPs in combination with antibiotics, adjust the ratio of hydrophilic and hydrophobic peptides to reduce toxicity to host cells, or optimize the length of peptides [23]. Finally, the AMPs can be produced on a large scale through technology for recombinant protein production using microorganisms such as *E. coli* and yeast [24].

The AMPs currently in clinical use include Polymyxins, LL-37, and Defensins. Polymyxins, used to treat infections such as *Pseudomonas aeruginosa* and *Acinetobacter baumannii*, are being studied to reduce their toxicity and increase efficacy [25]. LL-37, being studied as a treatment for skin infections and wound healing, is being developed with modifications and combination therapies to enhance its antibacterial effect [26]. Defensins, which have a wide range of antibacterial effects against bacteria, viruses, and fungi, are being used to increase their stability and reduce resistance [27]. Although the NB_AMP2 was identified to exhibit superior antimicrobial activity compared to other NB_AMPs in this study, it exhibited lower activity compared to the AMPs currently in clinical use, such as Polymyxins, LL-37, and Defensins. Therefore, the NB_AMPs need to be further improved in their functionality and large-scale productivity for future clinical applications, as described above.

## Figures and Tables

**Table 1:** Oligonucleotides of *N. radioresistens* applied for this study.

Forward		Reverse	
Oligonucleotide Name	Nucleotide Seq	Oligonucleotide Name	Nucleotide Seq
NB_AMP1-F	AAGTGGTGCGCGTCATAAAG	NB_AMP1-R	TCAAAGTCTGGCGTTTACGG
NB_AMP2-F	AGTCCATGATGTGCGTGAAC	NB_AMP2-R	ATCAGGTTGCAACGGCCTAA
NB_AMP3-F	GACGCCCAAAAAAGTTGACG	NB_AMP3-R	GCAAACATGATGGCTGCCTA
NB_AMP4-F	AGCCCTGAAAGCTCTATCAAAG	NB_AMP4-R	CAGCAAAGCAAAGCTGCTCA
NB_AMP5-F	GGCAACATAAGCTTCCTCTG	NB_AMP5-R	TGTATGAGATTGGAGCAGGA
NB_AMP6-F	ACTGGAAGCCGATGATCATG	NB_AMP7-R	TGGCTCATGCCTTTGATTGG
NB_AMP7-F	TTTACGACTACCTCACCACC	NB_AMP6-R	AGTTGGTGTATGTGGGCTTC
NB_AMP8-F	TTACGACCTCTCTTAGCTCC	NB_AMP8-R	TAACAGAATTGGGGACGCAC
NB_AMP9-F	CAGAGACGTGATTCCTTTCC	NB_AMP9-R	TTCATAGCGGTTGCGGTTAG
NB_AMP10-F	ATCCCAAAGACCACAGTACC	NB_AMP10-R	AAGAGCCGATGATGGTAGAC
NB_AMP11-F	AAGCTTATCATTACCCACCCTA	NB_AMP11-R	CTGACTGCAAATGCTGCAAC

**Table 2:** NB_AMPs of *N. radioresistens* applied for this study.

AMP Name	ORF Sequences *	Homologous AMPs		Synthesized AMPs			
		Homolog Name	Identities/ Positives (%)	Amino Acid Sequences	Amino Acid Number	pI	MW
NB_AMP1	MENHTGNTSSNRMMTGMFKDRESAERAYNALHSRGYSKDDVNVIMSDDARKRHFSDSHNNDTELGDKALEGAGAGSAIGGTLGAIVGAIAAIGTSVALPGLGLVIAGPLAAGLAGAGAG**GLTGGLLGALVGSG**IPEDRAKVYESGVKEGNIVMG	Leptoglycin	71/79	-	-	-	-
NB_AMP2	MAKRNKKYLESADPVCGLCEREVGFTTLHHLIPREEGGKHGPTVPLCQPCHSTIHLTYTNKELAVLYNNVHALRASE**GLQKYLSWVKNKRL**DKITNRRGKGNRKR	Melittin	57/71	GLQKYLSWVKNKRL	14	10.46	1733.09
NB_AMP3	*MKKITSIWLAAAFGFCMITSPLAA*QDTTKVQETNKEKAKHGTHQVGHGTKEVGKGTKKVVVAGAKATG**KGAKKAGKAVKKTVKK**GVDKVD	Cecropin-B1	50/64	KGAKKAGKAVKKTVKK	16	10.85	1670.12
NB_AMP4	MDTYNLKPENMRAPEHLNEAEARKSLEELDAKIKVLQGRAHATAADSHHTYHEHIAALEAKRALIAQKLENSTTATDST**WQEIKNSLEDLSDSI**KKLF	Caerin-4.3	53/73	WQEIKNSLEDLSDSI	15	3.92	1776.92
NB_AMP5	MTS**NTLSSIASLLKK**LISRLTGPTLQPIPVPVRQQPNR	H/V-peptide	67/83	NTLSSIASLLKK	12	10.00	1274.52
NB_AMP6	*MKDNQKDQQSSNLGSTTG*GSMGNTGASGSTGSGMGSSSSSSGMSSGSSGMSGSTGSMGSGTSGSGLSGGTSGSGMSGSSSGMSGSTGSTSKKGSSSLTSSKGTSGSSSTLSAGSTDKKSSTSKSSTPKASTSKSGSTSKSGKSSSSKSGSSASNSSKMGQDNDSMMGGQSDSMRNMGG**GQYNQGGYGSQGGDYGQGGYGQGSMGGGYG**	NLP-30	49/60	GQYNQGGYGSQGGDYGQGGYGQGSMGGGYG	30	3.80	2864.87
NB_AMP7	*MKDNQKDQQSSNLGSTTG*GSMGNTGASGSTGSGMGSSSSSSGMSSGSSGMSGSTGSMGSGTSGSGLSGGTSGSGMSGSSSGMSGSTGSTSKKGSSSLTSSKGTSGSSSTLSAGSTDKKSSTSKSSTPKASTSKSGSTSKSGKSSSSKSGSSASNSSKMGQDNDSMMGGQSDSMRNMGGGQYNQGGYGSQGGDYGQGGYGQGSMGGGYGQQHGES**WGQGGSNYGQGGYGSSMGGNMGGSNYGQGGYGGQGSMG**	NLP-29	51/55	-	-	-	-
NB_AMP8	MQIASVYTHQLVS**NYKLLSNSLNILL**KIEGICI	Hemoglobin subunit alpha	62/85	NYKLLSNSLNILL	13	8.59	1504.79
NB_AMP9	*MLKKYATSLLLVLALFVGSAQA*QSSQDKEKEKKELAKQKAAEKKAEGLAKAQAAKEKEKAKAAAIKEKEAAKKAADKEKAAAAKAKEQQKKEAAKQKALAAKEKEKAKAAEAKQKAAA**KKLAAKEKAAAAKEKEAAKKAAAKEKK**KA	DBAASP_471	56/67	KKLAAKEKAAAAKEKEAAKKAAAKEKK	27	10.08	2839.42
NB_AMP10	MSDRFRDTYRIPSARLQDWDYGWNAAYFVTICTKDKQH**FFGRFKKEK**WSCLRLDNWLKDFGKRFQPISHLFY	DBAASP_6715	78/100	FFGRFKKEK	9	10.29	1186.42
NB_AMP11	MTEETTLLKNYSDQEKGAYLGALATIASADGTVTEDELTFLRLLGEAAELPASLEQEVESIAKNPSQISLQKCLDVLKASDLRFSFVTDLISFAKSDGEYSPEEQQRIGEIGQYLGIDQKQFSILDQFVDKANQAQQQGEDPTSQSFLNKSGFGDMFKKSGISPGMVTGMLGILAPMVISGMMRRK**GGRSMGMGGGMMGGMGGGLGGLLGGLLGGGMMSRGGMYGGGRTGGLGSMASILGGLAGRSRYGGMGSGGLGGLLGGILGGGRRGGGTG**W	Acanthoscurrin-1	54/60	-	-	-	-

* The bold sequences represent putative AMP sequences, and the italic sequences with underlines in the NB_AMP3, 6, 7, and 9 indicate the signal sequences. The putative AMP and signal sequences were predicted using CAMP (http://www.camp.bicnirrh.res.in/campHelp.php, accessed on 15 April 2024) and SignalP 5.0 (https://services.healthtech.dtu.dk/services/SignalP-5.0/, accessed on 17 April 2024), respectively.

**Table 3:** Expression profiling of transcriptomes from *N. radioresistens*.

Sample	Total Reads	Mapped_Reads	Mapping_Rate%	Count (>0)	Exp (>1)
Nibribacter_1	69,515,047	11,295,952	16.25	3797	3797
Nibribacter_2	59,667,108	2,588,387	4.34	3797	3797

Nibribacter_1; exponential phase, Nibribacter_2; stationary phase; Reference; 3,797 genes from *N. radioresistens*.

**Table 4:** Expressions of candidate *AMPs* in *N. radioresistens*.

SeqName	AMP Name	Nibribacter_1.c	Nibribacter_2.c	Nibribacter_1.e	Nibribacter_2.e
>MLDJCCKF_00064	NB_AMP1	14,665.99	1832.39	2491.731	368.873
>MLDJCCKF_00258	NB_AMP2	838	828.57	142.375	166.797
>MLDJCCKF_00361	NB_AMP3	4062.06	1139.23	690.138	229.334
>MLDJCCKF_00464	NB_AMP4	2212.92	1031.13	375.972	207.572
>MLDJCCKF_01600	NB_AMP5	39,418.47	10,317.65	6697.144	2077.011
>MLDJCCKF_01708	NB_AMP6	1738.38	877.3	295.348	176.606
>MLDJCCKF_01884	NB_AMP7	6896.51	3234.09	1171.707	651.043
>MLDJCCKF_02400	NB_AMP8	136,561.28	4190.21	23,201.575	843.517
>MLDJCCKF_02565	NB_AMP9	1294.13	1079.39	219.871	217.289
>MLDJCCKF_02663	NB_AMP10	1105.5	542.04	187.824	109.116
>MLDJCCKF_02732	NB_AMP11	546.7	472.2	92.884	95.058

Nibribacter_1: exponential phase, Nibribacter_2: stationary phase, c: read count, e: normalized.

**Table 5:** MIC50s of synthetic NB_AMPs against *E. coli* and *S. aureus*.

Synthetic Peptides	NB_AMP2	NB_AMP3	NB_AMP4	NB_AMP5	NB_AMP6	NB_AMP8	NB_AMP9	NB_AMP10
*E. coli*	0.17 mg/mL (0.10 mmol/mL)	3.43 mg/mL (2.05 mmol/mL)	4.96 mg/mL (2.79 mmol/mL)	10.21 mg/mL (8.01 mmol/mL)	9.43 mg/mL (3.29 mmol/mL)	-	-	2.60 mg/mL (2.19 mmol/mL)
*S. aureus*	0.23 mg/mL (0.13 mmol/mL)	2.88 mg/mL (1.73 mmol/mL)	2.95 mg/mL (1.66 mmol/mL)	3.24 mg/mL (2.55 mmol/mL)	5.83 mg/mL (2.04 mmol/mL)	8.36 mg/mL (5.55 mmol/mL)	5.05 mg/mL (1.78 mmol/mL)	2.11 mg/mL (1.78 mmol/mL)

## Data Availability

The original contributions presented in this study are included in the article. Further inquiries can be directed to the corresponding author.
